# Quercetin-liposomes effectively regulated the Nrf2/Keap1 and NF-κB/P38 MAPK signaling pathways and protected the liver against paracetamol-induced damage

**DOI:** 10.1016/j.jgeb.2025.100617

**Published:** 2025-11-22

**Authors:** Fatma El Zahraa A. Elkady, Walaa A. Moselhy, Fatma I. Abo El-Ela, Abeer M. Abd El-Hameed, Mohamed I. Zanaty

**Affiliations:** a Biotechnology and Life Science Department, Faculty of Postgraduate Studies for Advanced Sciences, Beni-Suef University, Beni-Suef, Egypt; b Medical Laboratory Technology Department, Higher Technological Institute of Applied Health Sciences, Beni-Suef, Egypt; c Department of Forensic Medicine and Toxicology, Faculty of Veterinary Medicine, Beni-Suef University, Beni-Suef, Egypt; d Pharmacology Department, Faculty of Veterinary Medicine, Beni-Suef University, Beni-Suef, Egypt; e Department of Chemistry, Faculty of Science, Taibah University, P.O. Box 30002, Al-Madinah Al-Munawarah 14177, Saudi Arabia

**Keywords:** Quercetin, Liposomes, Paracetamol, Hepatotoxicity, Nrf2, Keap1, NF-κB, P38 MAPK

## Abstract

The prevalence of hepatotoxicity has sharply increased worldwide in recent decades. Our study aimed to enhance quercetin’s effectiveness against paracetamol (PCM)-induced hepatotoxicity using a quercetin liposome nano formulation. The quercetin liposome (QL) nano formula was fabricated by applying the thin-film hydration method. It was examined via dynamic light scattering, confirmed with a transmission electron microscope (TEM), and followed by assessments of drug-loading capacity, encapsulation efficiency (EE%), and Fourier transform infrared (FTIR) spectroscopy. The release features and cell cytotoxicity were also assessed. The in vivo parameters were completed. We effectively synthesized and characterized the quercetin liposome nanoformula with a 501.9 nm particle size and a –22.8 mV zeta potential. TEM imaging showed that quercetin liposomes were spherical. The EE% for the optimized formulation was 77.1 %. FTIR test confirmed the quercetin liposome spectra. Sustained release behavior of about 67.45 % of quercetin was released from liposomes by 24 h. The IC50 value was reduced from 71.32 µg/ml to 51.28 µg/ml for quercetin and quercetin-loaded liposomes. For the in vivo study, quercetin liposome improved all the altered biochemical markers, alleviating the levels of Malondialdehyde (MDA), Nitric oxide (NO), Superoxide dismutase (SOD), and Glutathione peroxidase (GPx), and upregulating Nuclear factor erythroid 2-related factor 2 (*Nrf2*) with a downregulation of Kelch-like ECH-associated protein 1 (*Keap1*), Nuclear factor kappa-light-chain-enhancer of activated B cells (*NF-κB*), and p38 Mitogen-activated protein kinase (*P38 MAPK*) gene expression that paralleled the histopathological amelioration. Our results suggested that the quercetin liposome nano formulation had potent hepatoprotective activity through ameliorating biochemical indicators, oxidative stress markers, upregulation of anti-apoptotic genes, and improvement in the histopathological index.

## Graphical abstract

**Figure:**
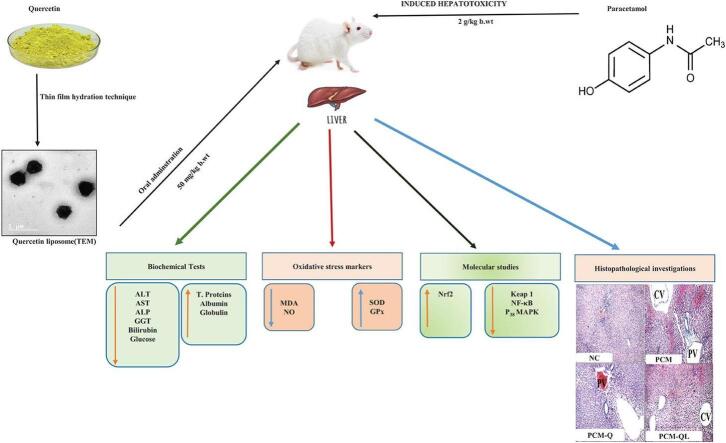

## 1 Introduction

Hepatotoxicity can result from prescription drugs, herbal medicines, natural substances, or any toxicant or poison, and it manifests as morphological and functional impairment [1]. Drug overdose can damage the liver, causing fibrosis, acute liver failure, and death in humans and animals. Approximately 1,000 pharmaceuticals, chemicals, botanical remedies, and dietary supplements adversely affect the liver in 50 % of acute liver failures [2], [3]. Over 75 % of unique medication reactions cause liver transplantation or death. Drug-induced liver injury (DILI) and its prevention are intensively explored to reduce harm [4].

Hepatotoxic reactions are caused by various pathways, including immune-mediated processes, mitochondrial damage, oxidative stress, and the disruption of bile acids and other hepatic transporters [5]. Paracetamol can be used safely to treat COVID-19 symptoms such as fever, headaches, and acute or persistent discomfort. However, due to potential liver damage at high paracetamol doses, alertness must be maintained [6]. The disparity between reactive oxygen species (ROS) and cell antioxidant capability contributes to PCM toxicity. Due to hepatic ischemia, necrosis, and apoptosis, ROS alter gene expression and provoke significant hepatic injury [7]. Plant-derived natural products, especially phenolic and flavonoid compounds, frequently serve as hepatoprotective therapies against oxidative stressors [8], [9]. Silymarin is frequently utilized in the treatment and management of hepatic ailments by regulating glutathione concentration within the cells [10]. Its low bioavailability and poor water solubility explain why it has little therapeutic use [11].

Quercetin, a polyphenolic flavonol found in many fruits and vegetables, has beneficial antioxidant properties that affect biological, pharmacological, and therapeutic activities [12], [13]. Quercetin's main drawbacks are its short biological half-life, low bioavailability, low chemical stability, and low water solubility [14]. To counteract these downsides, nanotechnology offers new solutions [15]. Various nanocarriers have been developed to enhance quercetin’s solubility and create delivery systems specific to particular tissues. Moreover, liposomes are efficient drug-delivery vehicles because their phospholipid composition closely resembles cell membranes, making them biocompatible and minimally toxic and increasing drug uptake in specific cells and tissues [16]. Furthermore, liposome-loaded drugs are shielded from physiological processes such as enzyme degradation, chemical inactivation, the immune system, and rapid plasma clearance, which enhances and prolongs their duration of action [17]. This study aims to synthesize and characterize nano-formulated quercetin and evaluate its efficacy against paracetamol-induced hepatotoxicity.

## 2 Materials and methods

### 2.1 Chemicals and drugs

Lecithin, cholesterol, sodium deoxycholate (SDC), dialysis bag (12–14 kDa), paracetamol, ethanol HPLC, chloroform HPLC, and quercetin were brought from Sigma-Aldrich, USA. Serum liver enzymes, bilirubin, total proteins, albumin, and plasma glucose concentration were tested with kits provided by Spin React (Barcelona, Spain). From an approved vendor, we obtained the remaining compounds, which were of analytical quality.

### 2.2 Preparation of quercetin liposome

Quercetin liposome nano formulas were synthesized via the thin film hydration technique as follows: 20 mg cholesterol, 200 mg lecithin, 38.82 mg sodium deoxycholate (SDC), and 10 mg quercetin were mixed and dissolved in 5 ml ethanol and 5 ml chloroform in a round-bottomed flask: The resultant organic solution was placed in a rotary evaporator and allowed to evaporate at 40 °C under reduced pressure for 10 min, or until a thin, perfectly dry film appeared. To hydrate the lipid layer in the flask, 5 ml of PBS (pH of 7.4) was added, and it was then allowed to spin at 100 rpm for 20 min while maintaining normal pressure. The prepared formula was placed in a bath sonicator for 1 h to shrink the liposomes and kept at 4 °C.

### 2.3 Characterization

#### 2.3.1 Zeta Sizer and zeta potential measurements

A ZS90 Zetasizer device (Malvern, UK) was utilized to assess the hydrodynamic size and the potential charge of the disseminated QL nanoparticles by dynamic light scattering (DLS) [18]. In the present study, the alteration in the Zeta potential of QL nanoparticles was quantified using a PBS pH 7.4 dispersion with a refractive index of 1.479. Three replicates (n = 3) were conducted for all investigated parameters.

#### 2.3.2 High Resolution- transmission electron microscopy (HR-TEM) Study

The surface topography and particle size of the nanoformula were investigated using a high-resolution transmission electron microscope (JEM 1400, Japan) running at 300 kV. This measurement was achieved by depositing a single droplet of the diluted vesicular dispersion onto the exterior of a copper grid coated with carbon. Following applying a 2 % phosphotungstic acid negative stain, the grid was permitted to dry at ambient temperature for 10 min before undergoing additional analysis [19].

#### 2.3.3 Entrapment efficiency percentage (EE %)

For forty-five minutes, the temperature outside was kept at 4 °C and the speed of rotation was set at 14,000 rpm; the quercetin liposome formulation was centrifuged to extract the free quercetin-containing supernatant. The amount of unbound quercetin was quantified with a UV–VIS spectrophotometer set to λ max = 378.5 nm. A triplicate of each measurement (n = 3) was performed. Following this equation, the quercetin entrapment efficiency (EE%) was computed [20]:

Equation no 1. EE% = $\frac{\mathrm{Totalamountofquercetin}-Freeamountofquercetin}{\mathrm{Totalamountofquercetin}}$ × 100

#### 2.3.4 FTIR analysis

FTIR provides interface analysis for nanoparticle functional group surface adsorption [21]. FTIR spectra were estimated by the Bruker Vertex 70 FTIR-FT Raman spectrophotometer. After mixing the dry sample and physical mixture (1  mg) with KBr powder (100  mg), the mixture was ground evenly and pressed into pellets. Spectra were collected at 25 °C and characterized by a width of 1  cm− [1] and 64 scans within the wave number frequency of 400–4000  cm− [1].

#### 2.3.5 Quercetin in vitro release

The dialysis bag approach was used to measure the amount of quercetin released from the quercetin liposome [22]. Glass cylinders with an internal diameter of 2.5 cm and a total length of 6 cm were filled with aliquots of quercetin nanodispersion, equivalent to 3 mg of quercetin, and their ends were adequately covered with a presoaked dialysis membrane with a cut-off molecular weight of 12,000 Da. The release medium was supplemented with 200 ml of PBS (0.01 % v/v Tween 80, pH 7.4). During the release experiment, to determine sink conditions, the ambient temperature was set at 37 ± 0.5 °C, and the speed of rotation was adjusted to 200 revolutions per minute at specific intervals of 1, 2, 4, 6, 8, and 24 h; 2 ml aliquots were taken out regularly. After that, they were recharged with a new medium of equal volume to ensure they had a similar volume to support a fixed total volume. A similar investigation examined the in vitro release profile of 1 ml of free quercetin. This was performed by preparing 1 ml of 3 mg/ml quercetin in PBS at pH 7.4 and 60 % v/v polyethylene glycol 400. A spectrophotometer measured the quercetin concentration at λ max = 378.5 nm. Every measurement was conducted three times.

### 2.4 *In vitro* cytotoxicity assay and IC50

The cytotoxicity experiment was conducted using Vero cell lines (ATCC, USA). We have selected Vero cells as a preliminary model for general cytotoxicity assessment due to their ease of culture, rapid growth, and common use in initial biocompatibility screening of nanomaterials. The cells were grown in DMEM media with streptomycin, penicillin, and 10 % FBS at concentrations of 100 mg/mL and 100 units/mL, respectively. Next, the trials were carried out in a controlled environment with a relative humidity of high levels and a carbon dioxide content of 5 % at a temperature of 37 °C. To make a stock solution, we diluted each sample in full DMEM at 37 °C. Six or seven concentrations were tested using two-fold serial dilutions. Confluent monolayers of cultivated cells were developed for 24 h in 96-well microtiter plates. There were three sets of test samples maintained in triplicate at 37 °C in a CO2 atmosphere for 48 h. Each well was heated for 4 h at 37 °C after receiving 20 μL of 5 mg/mL MTT after the initial incubation. Then, adding 150 μL of MTT solvent, carefully take out the medium. Place the cells in an orbital shaker and shake for fifteen minutes after covering them with tinfoil. The optical density of the microplate reader was finally measured at 570 nm [23], [24].

### 2.5 *In vivo* investigations

#### 2.5.1 Animals and experimental Design

We purchased four-week-old male albino rats from VACSERA in Egypt, weighing 150–200 g. For seven days, the rats were housed in specially designed, see-through cages that were kept at a constant 26 °C and had a light–dark cycle of 12:12 h. The University of Beni-Suef's Institutional Animal Care Committee (IACUC) approved the use of these animals for research (BSU/022–430).

There was a total of 30 rats divided into 5 groups, with 6 animals per group. The NC-group, which stands for “normal control,” received 1 ml/kg b. wt. of PBS orally for 14 successive days. The PCM-group, which consisted of animals given paracetamol, received PBS at a dosage of 1 ml/kg b. wt. for 14 days. On the 13th day, before the experiment's last day, they were given a hazardous dose of paracetamol orally, 2 gm/kg b. wt. [25] ]. The quercetin dosage for the paracetamol quercetin group (PCM-Q) was 50 mg/kg body weight [26]. The paracetamol quercetin liposome group (PCM-QL) received a quercetin liposome in a dose equal to 50 mg/kg body weight. The paracetamol silymarin group (PCM-S) received silymarin at a dose of 200 mg/kg body weight [27]. Animals took quercetin, quercetin liposome, and silymarin orally through a stomach tube for 14 days. Afterward, on the twelfth day before to the experimental end, a lethal dosage of paracetamol (2 gm/kg body weight) was administered. The doses were modified according to the fluctuations in the rats' body weight.

#### 2.5.2 Preparation of samples

At the end of the experiment (14th day) and after overnight fasting, all animals were given a ketamine/xylazine mixture with a dosage of 0.2 ml/100 g b.wt to induce anesthesia [28]. After that, blood samples were obtained from each rat; all rats were euthanized. The obtained blood samples were split into two tubes: the first, a plain one for serum analysis, and the second tube containing sodium fluoride for plasma glucose determination. Centrifugation was applied to the obtained blood samples at 4 °C and 4000 × g for 15 min. Separation and storage at −20 °C of the serum allowed for further biochemical analysis.

#### 2.5.3 Tissue homogenization

After the euthanasia of animals, the liver was extracted and cleaned with regular physiological saline and split into three sections. Section one involved homogenizing 0.5 g of liver tissue in 5 mL of pH 7.4 phosphate-buffered saline. The homogenates were then centrifuged at 4 °C for 20 min at 1200 rpm. Ultimately, the supernatants were separated and kept in storage at −80 °C for the determination of oxidative stress markers. The second section was embedded in 10 % neutral formalin for histological evaluation. To conduct the molecular test, a third part was frozen at −80 °C.

### 2.6 Antioxidant assay

Using test kits from Biodiagnostic Company, Dokki, Giza, Egypt, we assessed MDA, NO, SOD, and GPx in liver clear homogenate samples in the current experiment in compliance with the guidelines provided by the manufacturer.

### 2.7 Molecular assay

#### 2.7.1 Nrf2, Keap1, NF-κB, and P38 MAPK gene expression detection by RT- PCR

Cellixizol Reagent (CelliXiza Biotechnology Company): Following the manufacturer's guidelines, RNA was extracted from hepatic tissues. Then, complementary DNA production was carried out using the CelliXiza reagent (CelliXiza Biotechnology Company) as instructed by the manufacturer. For real-time PCR, a 20-μL system including one μL for each primer (forward & reverse) as indicated in Table 1, two μL of cDNA, ten μL of master mix, and six μL of DEPC water was utilized using a Step One Plus Real-Time PCR System (Applied Biosystems, Thermo Fisher Scientific, USA).

**Table 1:** Primers for RT- PCR.

**Gene**	**Forward**	**Reverse**
Nrf2	CATTTGTAGATGACCATGAGTCGC	ATCAGGGGTGGTGAAGACTG
Keap1	CTTCGGGGAGGAGGAGTTCT	CGTTCAGATCATCGCGGCTG
P38MAPK	AGAGTCTCTGTCGACCTGCT	CCTGCTTTCAAAGGACTGGT
NF-κB	TGGGACGACACCTCTACACA	GGAGCTCATCTCATAGTTGTCC
β-actin	TGACAGGATGCAGAAGGAGA	TAGAGCCACCAATCCACACA

The heat cycler was programmed to go through the following three stages: at the first stage, 5 min at 95 °C. The second stage continued for 40 cycles and included 95 °C for 30 s, 57–60 °C for 30 s, and 72 °C for 30 s. The final holding stage was set at 72 °C for 15 min. With normalization to β-actin, To examine the amplification data, the software provided by the manufacturer was used, and Schmittgen and Livak's 2^-ΔΔCt^ method was employed. [29].

### 2.8 Histopathological investigations

Small portions of livers were cut and immersed in formalin (10 %). Using hematoxylin and eosin, tissue samples were stained that were embedded in paraffin. The resultant histological alterations were examined under a light microscope[30].

### 2.9 Statistical tests

SPSS software (Chicago) (IBM SPSS for Windows 8, version 22) was implemented for statistical analysis. Duncan's test examined the data via ANOVA. Results are presented as mean ± standard error (SE), with a significance P value at P < 0.05.

## 3 Results

### 3.1 Characterization of quercetin liposome nanoformula

#### 3.1.1 Size and charge measurements

**Fig. 1:**
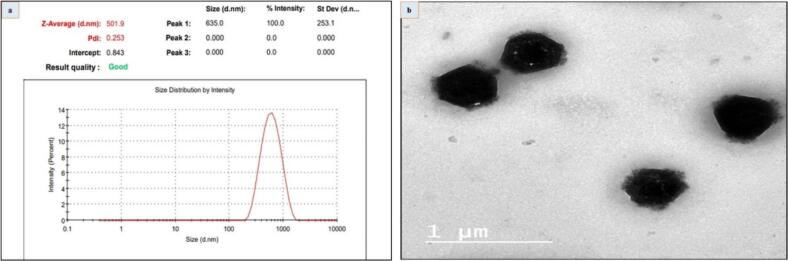
**(a)** Particle size, Polydispersity index (PDI) of quercetin liposomes (QLs) characterized by DLS, and (b) surface morphology of optimized quercetin liposomes (QLs) formulation by TEM.

The quercetin liposome vesicles in our investigation had an average size of 501.9 ± 1.58 nm and a polydispersity index (PDI) of 0.253, as shown in Fig. 1a. A value of –22.8 ± 0.53 mV was found for the quercetin liposome's zeta potential.

#### 3.1.2 Transmission electron microscope (TEM)

Based on the data that we have; it seems that the quercetin liposome nano formula is approximately spherical and has a very even distribution of sizes. The size of the particles varies between 450 and 550 nm, as seen in Fig. 1b.

#### 3.1.3 Entrapment efficiency percentage (EE %)

To measure the quantity of quercetin loaded into liposomes, the EE% of the formulation that was created was examined. The amount of quercetin contained within the liposomes was determined to be 77.1 % using equation No. 1.

#### 3.1.4 FTIR determination

**Fig. 2:**
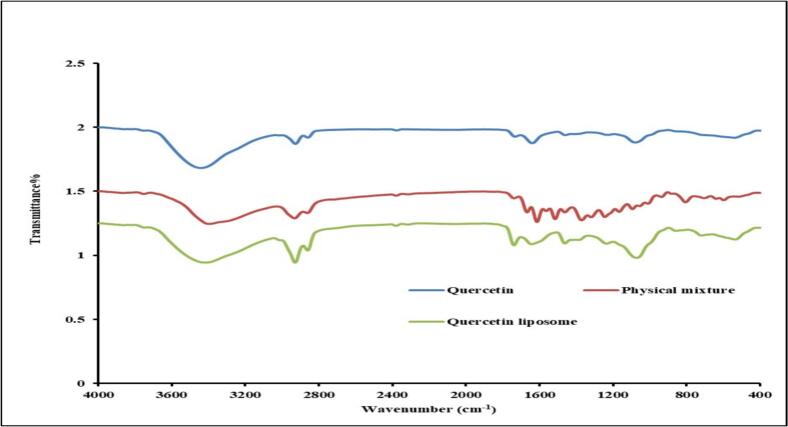
Fourier-transform infrared spectroscopy (FTIR) of quercetin, physical mixture, and quercetin liposome.

Fig. 2 illustrates the FTIR data for both crude quercetin and quercetin liposome nano formulas. The FTIR spectrum of crude quercetin displays distinct peaks attributed to various functional groups, including 3389 cm^–1^, 1666 cm^–1^, 1611 cm^–1^, 1511 cm^–^ [1], and 1242 cm^–^ [1], which correlate to O–H, C=O, aromatic C=C, and C-O stretching vibrations, respectively. Similar characteristic peaks are identified in the quercetin liposome spectra with variations in the intensities.

#### 3.1.5 Quercetin in vitro release

**Fig. 3:**
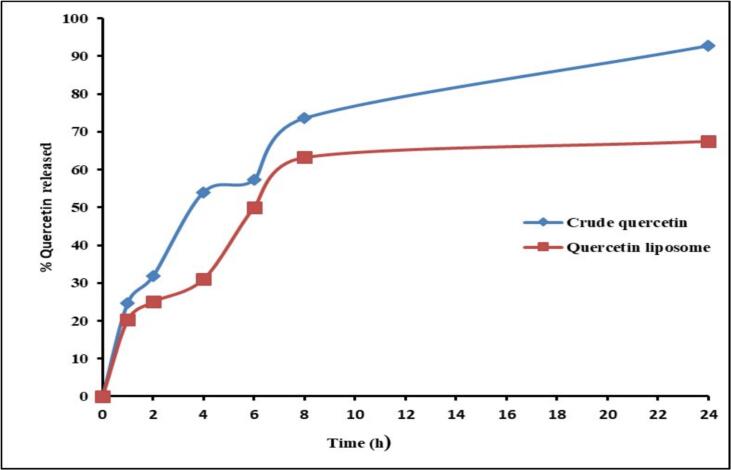
Comparative in vitro drug release profile of crude quercetin and quercetin liposomes.

The release of quercetin from the quercetin liposome was demonstrated over 24 h at 37 ± 0.5 °C, pH 7.4 (Fig. 3). The initial release rate of free quercetin and quercetin liposomes varied during the first hour of shaking, with a cumulative release percentage of approximately 92.75 % after 24 h for the crude quercetin. Meanwhile, the quercetin liposome exhibited a slow-release rate and an extended-release pattern, with a cumulative release of 67.47 % at the end of the experiment.

### 3.2 *In vitro* cytotoxicity

**Fig. 4:**
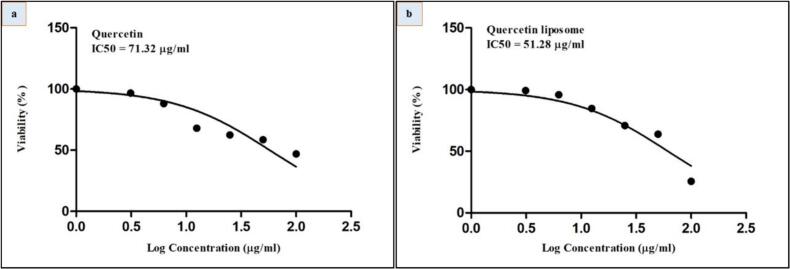
Cell viability for Vero Cell Lines using MTT cytotoxicity assay. Treatment of cells with quercetin liposomes (QLs) effectively reduced the IC_50_ value from 71.32 μg to 51.28 μg.

Regarding cell cytotoxicity, our study revealed that both high and low concentrations of quercetin and quercetin liposomes had no cytotoxic effects on normal cells. Quercetin liposomes effectively reduced the IC50 value from 71.32 μg to 51.28 μg, as indicated in Fig. 4.

### 3.3 *In vivo* study in hepatotoxic rat model

#### 3.3.1 Effect of quercetin liposome nano formula on serum liver enzymes and bilirubin

**Fig. 5:**
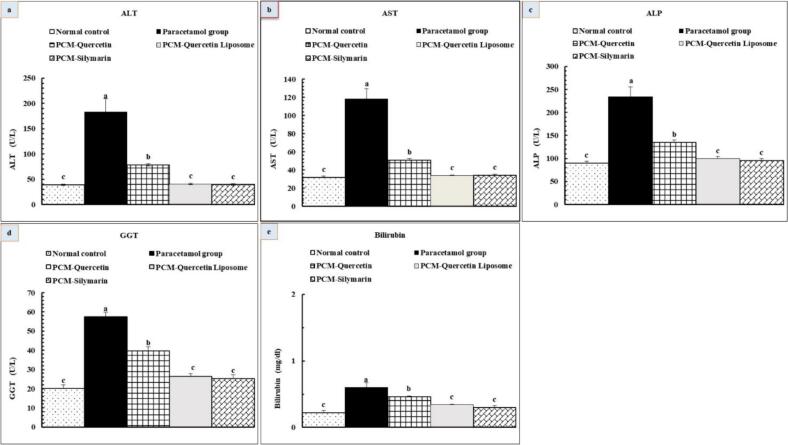
Effects of quercetin and quercetin liposomes on liver function tests: (a) ALT, (b) AST, (c) ALP, (d) GGT, and (e) Bilirubin. Data are expressed as mean ± standard error (n = 6), means which share the same superscript symbol (s) are not significantly different, P ≤ 0.05.

Our data proved that the PCM-treated group revealed a notable elevation in serum ALT, AST, ALP, GGT, and bilirubin concentrations (P < 0.05) compared to the normal control group. On the contrary, treatment with quercetin and quercetin liposome induced a substantial improvement in all liver enzymes and bilirubin levels (P < 0.05), with the most potent efficiency of the QL nanoformula, as represented in Fig. 5.

#### 3.3.2 Effect of quercetin liposome nanoformula on total proteins, albumin, fasting plasma glucose levels, and body weight

Our present data show that total protein and albumin levels were notably reduced after PCM induction (P < 0.05) and markedly increased in the quercetin and quercetin liposome groups (P < 0.05). In addition, as compared to the normal control group, our data suggest that the PCM group exhibits a significant increase in fasting plasma glucose levels (P < 0.05). In contrast, treatment with quercetin and quercetin liposome exhibits a substantial drop in fasting plasma glucose levels and a marked increase in total protein and albumin levels (P < 0.05). The present data reveal that the quercetin liposome nanoformula is more effective than quercetin alone in ameliorating all the altered liver biomarkers, as represented in Table 2.

**Table 2:** Effects of quercetin and quercetin liposomes on total proteins, albumin, globulin, fasting plasma glucose levels, and body weight.

**Parameter** **Groups**	**Total Proteins** **(g/dl)**	**Albumin** **(g/dl)**	**Globulin** **(g/dl)**	**Fasting glucose** **(mg/dl)**	**Initial Weight (gm)**	**Final Weight** **(gm)**
**NC-group**	5.6 ± 0.12 ^a^	3.52 ± 0.058 ^a^	2.08 ± 0.003^b^	101.2 ± 4.01^c^	158 ± 2.55^b^	174 ± 2.92^b^
**PCM-group**	4.7 ± 0.07^c^	2.79 ± 0.029^c^	1.91 ± 0.005 ^d^	156.2 ± 5.86 ^a^	136 ± 1.87^c^	149 ± 1.87^c^
**PCM-Q**	5.1 ± 0.07^b^	3.06 ± 0.027^b^	2.04 ± 0.002^c^	136.4 ± 1.86^b^	166 ± 7.97^b^	184 ± 8.12 ^ab^
**PCM-QL**	5.54 ± 0.08^a^	3.4 ± 0.083 ^a^	2.14 ± 0.002 ^a^	114.8 ± 10.42^c^	158 ± 7.52^b^	200 ± 10 ^a^
**PCM-S**	5.56 ± 0.06 ^a^	3.42 ± 0.086 ^a^	2.14 ± 0.003 ^a^	113 ± 5.98^c^	187.2 ± 8.73 ^a^	205 ± 8.06 ^a^
**F-Prob.**	P < 0.0001	P < 0.0001	P < 0.0001	P < 0.0001	P < 0.0001	P < 0.0001

Data are expressed as mean ± standard error (n = 6), means which share the same superscript symbol (s) are not significantly different, P ≤ 0.05. Normal control group (NC-group); Paracetamol group (PCM-group); Paracetamol quercetin group (PCM-Q); Paracetamol quercetin liposome group (PCM-QL); Paracetamol silymarin group (PCM-S).

#### 3.3.3 Effect of quercetin liposome on oxidative stress indicators

**Fig. 6:**
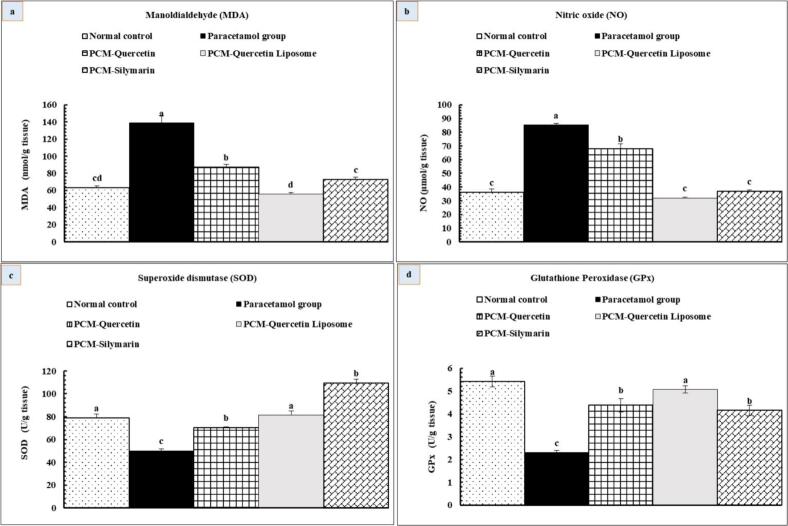
Effects of quercetin and quercetin liposomes on oxidative stress markers: (a) MDA (b) NO (c) SOD and (d) GPx. Data are expressed as mean ± standard error (n = 6), means which share the same superscript symbol (s) are not significantly different, P ≤ 0.05.

The PCM group exhibited a marked elevation (P < 0.05) in MDA and NO concentrations compared to the normal control group. Moreover, the PCM group displayed a notable reduction (P < 0.05) in SOD and GPx levels relative to the normal control group. Conversely, treatment with quercetin and quercetin liposome resulted in a substantial increase (P < 0.05) in SOD and GPx concentrations, along with a notable reduction (P < 0.05) in NO and MDA activities compared to the PCM group. Quercetin liposome therapy is more effective than free quercetin in ameliorating oxidative stress markers, as shown in Fig. 6.

#### 3.3.4 Effect of quercetin liposome nanoformula on Nrf2, Keap 1, and NF-κB and P_38_ MAPK gene expression

**Fig. 7:**
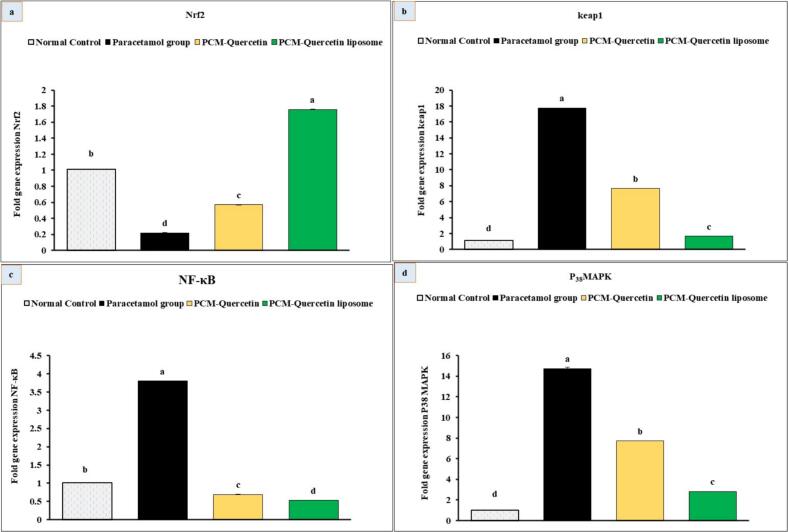
Effects of quercetin and quercetin liposomes on gene expression of (a) Nrf2, (b) Keap-1, (c) NF-κB and (d) p_38_ MAPK in the liver tissue. Data are expressed as mean ± standard error (n = 6), means which share the same superscript symbol (s) are not significantly different, P ≤ 0.05.

When comparing the PCM group to the normal control group, Fig. 7 reveals that PCM significantly decreased Nrf2 levels (P < 0.05) and increased Keap1 mRNA levels (P < 0.05). Contrarily, as compared to the PCM group, the quercetin and quercetin liposome groups showed a significant decrease (P < 0.05) in Keap1 levels and a significantly higher Nrf2 level (P < 0.05) after treatment. In addition, compared to the normal control group, the PCM group showed higher expression of both NF-κB and P38 MAPK. The levels of NF-κB and P38 MAPK were significantly lower (P < 0.05) in the quercetin and quercetin liposome groups when contrasted with the PCM group.

### 3.4 Histopathological investigations

**Fig. 8:**
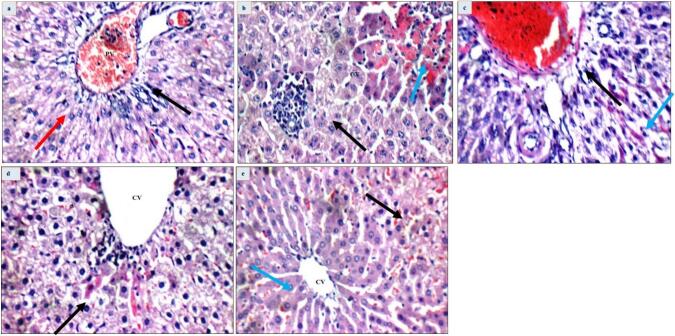
Histopathology images of rat's livers among different treatment groups (H&E X 400): (a) Normal control: high power view showing average portal tracts (black arrow) with average portal veins (PV), and average hepatocytes in the *peri*-portal area (red arrow) (H&E X 400); (b) PCM group: mild intra-lobular inflammatory infiltrate (black arrow), and markedly necrotic hepatocytes (blue arrow) (H&E X 400);(c) PCM − Quercetin: high power view showing average portal tracts (black arrow) with mildly congested portal veins (PV), and scattered apoptotic hepatocytes in the *peri*-portal area (blue arrow) (H&E X 400) (d) PCM – quercetin liposome: mildly dilated central vein (CV) with markedly apoptotic hepatocytes in the *peri*-venular area (black arrow) (H&E X 400) and (e) PCM- Silymarin: average central vein (CV), with mildly congested blood sinusoids (black arrow), and average hepatocytes in the *peri*-venular area (blue arrow) (H&E X 400).

Histological investigations revealed that liver specimens from the normal group showed normal characteristic features, as indicated in Fig. 8a. The PCM group had perivenular necrotic hepatocytes, minor intralobular inflammatory infiltration, and dilated central veins, which displayed a detached lining in their liver sections (Fig. 8b). Quercetin-treated hepatotoxic rats had average portal tracts with slightly dilated central veins, congested portal veins, and *peri*-venular apoptotic hepatocytes (Fig. 8c). Fig. 8d shows that quercetin liposome therapy ameliorated the altered structures of the liver sections, represented by the average size of hepatocytes in the area around the veins, portal tracts, and central veins, with slightly congested blood sinusoids. As anticipated, silymarin also exhibited considerable improvement in the liver tissues (Fig. 8e).

## 4 Discussion

Drug-induced hepatotoxicity is a condition resulting from short- or long-term use of natural or synthetic substances [31]. Several molecular mechanisms initiate hepatotoxicity, affecting metabolic pathways in exposed cells, such as mitochondrial poisons that block electron transport chain enzymes, resulting in the deterioration of fatty acid ß-oxidation (FAO) and inducing inflammation, steatohepatitis, and cirrhosis [32], [33], [34]. PCM was commonly recommended as a primary analgesic for COVID-19 individuals without considering the likelihood of concurrent toxicities [35]. Despite its safety, paracetamol overdose can induce severe hepatotoxicity, which is a major public health issue [36]. Recently, considerable emphasis has been directed at the defensive function and mechanisms of action of bioflavonoids, which possess antioxidant and anti-inflammatory characteristics and are readily available for dietary use [37].

Quercetin, a flavanone glycoside, is a highly physiologically active molecule within the flavonoid family, present in elevated concentrations in citrus fruits and vegetables. By scavenging oxygen radicals, protecting against lipid peroxidation, and chelating metal ions, quercetin demonstrated a wide range of pharmacological effects, including antioxidant and anti-carcinogenic actions that reduced oxidant damage and cellular apoptosis [38], [39], [40]. Meanwhile, the limited water solubility and low absorption of quercetin restrict its therapeutic in vitro applications [41]. Modern drug delivery systems have been established to maximize drug bioavailability and decrease its off-target accumulation by accelerating its delivery [42], [43]. Liposomes are superior to conventional drug delivery methods, consisting of a lipid bilayer that resembles the human cell membrane structure, which resulted in increasing its biocompatibility and minimizing its toxicity level [44], [45].

Our results indicated that quercetin liposome nano formulas were well-prepared and characterized by a spherical shape and a homogeneous size distribution. Li et al. and Munot et al. conveyed that liposome displayed a spherical shape and intact morphology characterized by non-detectable aggregation [46], [47].The particle size is a crucial parameter to consider when preparing nanoformulations, as it directly impacts their stability, release characteristics, and bioavailability, along with various other properties, such as absorption and accumulation in the liver, diffusion in tissues, extravasation, and renal excretion [48], [49], [50].

Nanoformulated drug characterization is particularly successful using dynamic light scattering (DLS) and high-resolution transmission electron microscopy (HR-TEM) [51]. DLS and HR-TEM differ in nanoparticle size measurement principally due to their fundamental principles and techniques. Using DLS, the average hydrodynamic diameter of quercetin liposomes was 501.9 ± 1.58 nm, which was somewhat larger than HR-TEM (450 nm). HR-TEM determines the actual diameter of the synthesized nanoparticles, whereas DLS determines their hydrodynamic diameter [52]. DLS utilizes light scattering to ascertain hydrodynamic diameters [53]. Conversely, HR-TEM offers high-resolution imagery of nanoparticles, facilitating direct observation and determination of their dimensions [53].

By contrast, the measurement of zeta potential provides important information for the surface charge of the particles [54]. The prepared QL nanoformula potential charge was –22.8 mV indicating that the prepared nanovesicles' were physically stable and considered a good carrier for oral drug delivery. Previous studies confirmed that the high zeta potential values increase the electrostatic repulsion, and propose creating a stable colloidal suspension that lessens nanoparticle agglomeration., which facilitates the creation of non-agglomerated suspension [55], [56]. In addition, the polydispersity index (PDI) of the QL nano formula was found to be 0.253, demonstrating that the nanoparticles were homogenous and monodisperse. Moreover, PDI assesses the homogeneity of formulations where values close to zero indicate uniform vesicle sizes and values that approach one reveal a wide variation in particle sizes, signifying a highly polydisperse nature [57].

Our present results indicate that the percentage of quercetin entrapped within liposomes is 77.1 %. The lipophilicity of quercetin facilitates its encapsulation within the bilayer membrane of the liposome. Our data agree with He et al. and Mohammadi et al., who stated that lipophilic substances typically exhibit higher EE when prepared using a vesicle bilayer [58], [59]. Meanwhile, improving the encapsulation of the drug within liposomes is a crucial factor in increasing its oral bioavailability [60].

FTIR examination was conducted to investigate the potential chemical bonding between quercetin and liposomes. Our results indicated that the spectra of quercetin liposomes showed the same characteristic peaks of quercetin, with slight variations in intensities due to the formulation of the liposomes. We suggest that quercetin interacted with the liposome bilayer via intermolecular hydrogen bonding [61].

Drug release is the movement of drug solutes from their material system to the release media [62]. The in vitro release experiment was done using a pH 7.4 PBS solution. The experiment's findings indicated a significant difference in the release patterns of quercetin and quercetin-loaded liposomes. Moreover, the drug release of free Q and QL was approximately 92.75 % and 67.47 %, respectively, within 24 h at pH 7.4. In addition, the release profile of QL was shown to be lower and slower in comparison to free quercetin, indicating the extended drug release characteristic pattern of liposomes [63]. Liposomes are regarded as very promising formulations for improving the bioavailability of drugs, exhibiting stable chemical and physical properties that result in a wide range of biomedical applications [64].

Cytotoxicity tests were performed to identify any evident side effects and learn more about the potential hazards of oral quercetin and quercetin liposome treatments. Regarding cytotoxicity, the present data indicated that quercetin liposomes effectively reduce the IC50 value from 71.32 μg to 51.28 μg. In the same line, Saraswat and Maher reported that the cytotoxicity level of quercetin was reduced upon liposomal encapsulation, which resulted in improving its therapeutic efficacy [65].

Liver enzyme functions and serum bilirubin levels are widely employed as the predominant biochemical indicators for assessing liver damage. Concerning the liver function panel, serum levels of bilirubin and liver enzyme activity were significantly elevated during PCM-induced liver damage, according to our results. Our data aligned with Abdel-Azeem et al., who reported that PCM induced a marked increase in liver enzyme activity [66]. One possible explanation for this effect is that paracetamol undergoes metabolism by cytochrome P450 enzymes, leading to the synthesis of N-acetyl-p-benzoquinone imine (NAPQI). In addition, NAPQI lowers glutathione levels in cells and causes protein adducts to form, especially in the mitochondria. This leads to dysfunction in the mitochondria, oxidative stress, and cell damage in the liver. As a result, these enzymes and bilirubin leak into the bloodstream, which is an indication of hepatic necrosis [67], [68]. Meanwhile, the administration of quercetin and quercetin liposome notably improved the altered enzymes and bilirubin levels in this study. Furthermore, the quercetin liposome formula enhanced the pharmacological efficiency of quercetin, protected the hepatocellular membrane, and preserved its structure, preventing intracellular enzyme leakage into the bloodstream. This effect supports the widely accepted belief that blood transaminases and bilirubin levels return to their normal state as the liver tissue heals and hepatocytes regenerate [69].

The results of this study demonstrated that serum albumin and total protein levels were drastically decreased following PCM overdose. The parenchymal cells of the liver predominantly synthesize albumin and several coagulation factors, as well as the majority of α-globulins and β-globulins, which are the principal sources of serum proteins [70]. A possible explanation for the decline in total protein and albumin concentrations following PCM administration is a reduction in the total number of hepatocytes that produce albumin, caused by necrosis [71]. Furthermore, previous studies reported that hepatic dysfunction leads to disturbances in both the qualitative and quantitative production of proteins, causing hypoproteinemia, which can alter animal physiological conditions [72], [73].

On the contrary, the quercetin and quercetin liposome pretreatment exhibited a notable elevation in the concentration of total proteins and albumin, indicating an enhanced process of protein synthesis. Consistent with our findings, previous data reported that paracetamol-treated rats exhibited a greater elevation of total protein and albumin concentrations after quercetin treatment [74], [75]. Moreover, our prepared quercetin liposome nanoformula increases the bioavailability of quercetin, maximizing its hepatoprotective effect through the notable amelioration of liver enzymes, bilirubin levels, and enhancement of protein production compared to crude quercetin.

Additionally, the present data show that fasting plasma glucose levels are elevated in paracetamol-induced hepatotoxic rats. Previous studies report abrupt hyperglycemia from paracetamol overdose. This is commonly due to concomitant hepatotoxicity. The cause is unknown, but paracetamol or its metabolites may directly impact the pancreas or the stress hormone response. Following our data, Massart et al. demonstrated that paracetamol induction led to glycogen and glutathione depletion, which correlates with hyperglycemia [76]. Additionally, MacFie et al. reported that substantial modifications in pancreatic beta cell ultrastructure may contribute to glycemic disorders [77]. Furthermore, hyperglycemia-induced hyper-inflammatory immune activation in Kupffer cells resulted in hepatic ischemia and reperfusion injury [78]. Conversely, quercetin and quercetin liposomes decreased plasma glucose levels. Our study's findings support the conclusions of M. Eid and S. Haddad, who proposed that quercetin's antidiabetic effects stem from the compound's capacity to reduce glucose absorption in the intestines and increase glucose utilization in the body's peripheral tissues. [79]. The findings showed that quercetin liposomes had a greater hypoglycemic effect than free quercetin in hepatotoxic rats.

Paracetamol primarily damages the liver by increasing reactive oxygen species, which occur when glutathione stores fall and the body's antioxidant defenses weaken [80]. To determine the toxicity induced by paracetamol, antioxidant factors, and oxidative stress indicators were assessed [81].The present study revealed a notable elevation in MDA and NO levels in the paracetamol group, whereas SOD and GPx values were notably decreased. These results align with the conclusions of El Fadil et al. who demonstrated that the innate antioxidant defense mechanism of the body is depleted due to an excess of free radicals and ROS produced under the administration of paracetamol [82]. Moreover, the induction of paracetamol can trigger lipid peroxidation and promote the formation of peroxynitrite, resulting in enhanced synthesis of nitric oxide (NO). Furthermore, peroxynitrite rapidly interacts with cell membrane lipids, resulting in potential adverse effects. Previous studies also reported higher levels of MDA and NO following PCM administration [83]. In addition, PCM induction resulted in a marked decrease in GSH concentration with notable prevention of antioxidant markers [69].

However, after pretreatment with quercetin and quercetin liposomes, MDA and NO levels dropped significantly, whereas SOD and GPx concentrations spiked. Previous research has demonstrated that quercetin can decrease elevated levels of MDA and NO in the paracetamol group, along with a notable increase in antioxidant markers. This suggests that quercetin might have antioxidant effects and protect tissues against lipid peroxidation [84], [85]. Additionally, quercetin's flavonoid fraction protects against lipid and protein oxidation and scavenges oxygen radicals; it also scavenges hydroxyl, superoxide, alkoxyl, and peroxyl radicals as it interacts with them [69]. Moreover, quercetin improves liver injury by increasing antioxidant enzyme levels [87]. Meanwhile, in quercetin-treated rats, SOD activity was elevated, potentially attributable to the enhancement of GPx activity that reduces H2O2 levels, thus averting *retro*-inhibition of SOD. These results align with prior research indicating a notable increase in SOD, GPx, GST, and CAT levels in paracetamol-treated rats following quercetin administration [69]. Nevertheless, quercetin liposome therapy is more effective than free quercetin due to its enhanced bioavailability and stability.

In our study, we investigated the mechanistic pathways of Nrf2/Keap1 and NF-κB/P38 MAPK to clarify the molecular concept behind PCM's hepatotoxic behavior and the pharmacological action of our formulation against its toxic activity. Our present data indicated that PCM induced a marked downregulation of Nrf2 expression with a notable upregulation of Keap1 expression in hepatic tissue. In contrast, treatment with free quercetin and quercetin liposome exhibited a different behavior by increasing Nrf2 expression and downregulation of Keap1 significantly. Our results followed the prior data that proved the cytotoxic impact of PCM on hepatocytes and its toxic potency against the Nrf2-Keap1 pathway [86]. PCM induces the synthesis of NAPQI, which rapidly depletes liver GSH. Moreover, free NAPQI interacts with protein sulfhydryl groups to create PCM adducts, losing Keap1-Nrf2 physiological control [87]. Oxidative stress is believed to be the fundamental mechanism behind paracetamol-induced cell death [88]. Moreover, nuclear translocation and protein activity were both altered after PCM induction [89]. This occurs because the rapid rate at which the medication is activated surpasses the cell's ability to defend itself by destroying essential proteins. However, quercetin liposome and free quercetin treatment greatly enhanced the Nrf2-Keap1 pathway. The redox balance inside cells is regulated by Nrf2 and its inverse regulator, Keap1. This balance affects inflammatory responses, hepatocellular mortality, and oxidative stress caused by PCM [90]. Overproduction of Nrf2 can reduce free radicals and strengthen antioxidant defenses, hence reducing harmful effects resulting from oxidative stress and critically regulating antioxidant and cytoprotective genes [91], [92]. Moreover, the present results proved that the cellular uptake and bioavailability of quercetin were improved by using the quercetin liposomal nano formula, which maximized the production of Nrf2 and its related cytoprotective genes.

For NF-κB/P38 MAPK pathways, the present results show that PCM induced a notable upregulation of NF-κB and P38 MAPK gene expression in hepatic tissue. In contrast, treatment with quercetin and quercetin liposome produced a notable downregulation in NF-κB and P38 MAPK expression. Consistent with other research, our findings show that NF-κB and P38 MAPK expression was considerably increased following PCM induction [81], [93]. Additionally, hepatocyte mortality caused by reactive oxygen species occurs when the MAPK and NF-κB pathways are activated [94]. Triggering the creation of inflammatory mediators is a crucial role of the NF-κB pathway. Increased levels of tumor necrosis factor-alpha, inducible nitric oxide synthase (iNOS), and cyclooxygenase-2 (COX-2) result from the activation of NF-κB binding motifs located in the promoters of these mediators [95].

INOS overexpression activates cell signaling pathways to promote NO generation and inflammation. In addition, the subsequent activation of MAPK under oxidative stress is essential in the intracellular signaling cascade of paracetamol toxicity [96]. On the other hand, quercetin and quercetin liposomes display the capacity to mitigate oxidative stress levels and effectively suppress the NF-κB and p38 MAPK pathways; they are key factors in oxidative stress and inflammation. Following our data, previous studies reported that the reduction in the oxidative markers, NF-κB, and p38 MAPK expression levels plays a critical role in assessing the efficiency of possible treatments for inflammatory diseases [97]. Compared to quercetin, the QL nano formula demonstrates superior attributes, including targeted delivery, prolonged half-life, controlled release, reduced systemic side effects, protection against degradation and clearance, and enhanced biocompatibility, thereby augmenting quercetin's therapeutic advantages.

Histopathological examination revealed that the liver samples from the PCM-intoxicated rats showed a mild dilatation in portal veins, slight inflammatory infiltration in the *peri*-portal area, markedly necrotic hepatocytes in the *peri*-venular area, and significantly dilated central veins with detachment in their lining. El Fadil et al. found that rats intoxicated with PCM had changes in their liver structure as a result of the making of ROS [82]. Quercetin-loaded liposomes improved these alterations. Moreover, QL can mitigate the overproduction of ROS due to its high antioxidant efficiency, suggesting that quercetin liposome nanoformulas have a greater ameliorative histopathological impact.

## 5 Conclusion

In summary, the present study concluded that quercetin, in a well-characterized form measuring 501.9 nm in size, was effectively encapsulated within a liposome with an entrapment efficacy of 77.1 %. The QL nanoformula significantly protects rats from PCM-induced liver damage. The prepared formulation alleviated biochemical liver functions and improved the oxidative stress condition by reducing SOD and GPx levels. Additionally, the reduction of Keap1, NF-kB, and P38 MAPK gene expressions, along with the upregulation of Nrf2, indicates the highly anti-apoptotic activity of the prepared formula. Consequently, the histopathological index improved. The prepared QL nano formula is an innovative strategy for the prophylactic management of PCM-induced liver damage.

**Ethical Approval**

“The use of these experimental animals was accepted by Beni-Suef University, Egypt's Institutional Animal Care Committee (IACUC) (BSU/022–430).”.

**Consent to Participate**

Not applicable.

**Consent to Publish**

Not applicable.

## CRediT authorship contribution statement

**Fatma El Zahraa A. Elkady:** Writing – review & editing, Writing – original draft, Visualization, Software, Resources, Methodology, Investigation, Formal analysis, Data curation. **Walaa A. Moselhy:** Writing – review & editing, Supervision, Conceptualization. **Fatma I. Abo El-Ela:** Writing – original draft, Visualization, Methodology, Investigation, Formal analysis, Conceptualization. **Abeer M. Abd El-Hameed:** Writing – original draft, Software. **Mohamed I. Zanaty:** Writing – review & editing, Visualization, Validation, Supervision, Software, Methodology, Formal analysis, Conceptualization.

## Funding

“The authors declare that no funds, grants, or other support were received during the preparation of this manuscript”.

**Availability of data and materials**

All data generated or analysed during this study are included in this published article. Should raw data files be needed in another format, they are available from the corresponding author upon reasonable request.

**Authors contributions**

All authors contributed to the study's conception and design. Material preparation, data collection, and analysis were performed by [Fatma El Zahraa A. Elkady] and [Mohamed I. Zanaty]. [Fatma I. Abo El-Ela], [Fatma El Zahraa A. Elkady], and [Mohamed I. Zanaty] performed the in vivo investigations. The first draft of the manuscript was written by [Fatma El Zahraa A. Elkady], [Mohamed I. Zanaty], [Walaa A. Moselhy], and [Abeer M. Abd El-Hameed], all of whom commented on previous versions. All authors read and approved the final manuscript.

## Declaration of competing interest

The authors declare that they have no known competing financial interests or personal relationships that could have appeared to influence the work reported in this paper.

## References

[1] Mihajlovic M., Vinken M. (2022). Mitochondria as the target of hepatotoxicity and drug-induced liver injury: molecular mechanisms and detection methods. Int J Mol Sci.

[2] Aithal GP, Kulkarni A V. Drug-induced liver injury. Medicine 2023;51:342–6.

[3] Porceddu M., Buron N., Roussel C., Labbe G., Fromenty B., Borgne-Sanchez A. (2012). Prediction of liver injury induced by chemicals in human with a multiparametric assay on isolated mouse liver mitochondria. Toxicological Sciences.

[4] Rani J., Dhull S.B., Rose P.K., Kidwai M.K. (2024). Drug-induced liver injury and anti-hepatotoxic effect of herbal compounds: a metabolic mechanism perspective. Phytomedicine.

[5] Miller RT. Risk Assessment for Hepatobiliary Toxicity Liabilities in Drug Development 2023. 10.1177/01926233231223751 38243687

[6] Dong E., Du H., Gardner L. (2020). An interactive web-based dashboard to track COVID-19 in real time. Lancet Infect Dis.

[7] Cichoż-Lach H., Michalak A. (2014). Oxidative stress as a crucial factor in liver diseases. World Journal of Gastroenterology: WJG.

[8] Salehi A., Hosseini S.M., Kazemi S. (2022). Antioxidant and anticarcinogenic potentials of propolis for dimethylhydrazine-induced colorectal cancer in Wistar rats. Biomed Res Int.

[9] Menon S., Al-Eisa R.A., Hamdi H. (2023). Protective effect of Annona muricata Linn Fruit Pulp Lyophilized Powder against Paracetamol-Induced Redox Imbalance and Hepatotoxicity in Rats. Processes.

[10] Oyman A., Unsal G., Aydogdu N., Usta U. (2022). Protective Effects of Silymarin on Acetaminophen-Induced toxic Hepatitis. EURASIAN JOURNAL OF MEDICAL ADVANCES.

[11] Kesharwani S.S., Jain V., Dey S., Sharma S., Mallya P., Kumar V.A. (2020). An overview of advanced formulation and nanotechnology-based approaches for solubility and bioavailability enhancement of silymarin. J Drug Deliv Sci Technol.

[12] Eldin O.S., Bakry S., Shaeir W.A.A., Mohammed M.S., Abd-Alzaher O.F.A. (2015). Possible hepatoprotective effect of quercetin against 2-butoxyethanol induced hepatic damage in rats. Middle-East J Sci Res.

[13] Yu X., Xu Y., Zhang S. (2016). Quercetin attenuates chronic ethanol-induced hepatic mitochondrial damage through enhanced mitophagy. Nutrients.

[14] Kaur S., Goyal A., Rai A., Sharma A., Ugoeze K.C., Singh I. (2023). Quercetin nanoformulations: recent advancements and therapeutic applications. Advances in Natural Sciences: Nanoscience and Nanotechnology.

[15] Teng H., Zheng Y., Cao H., Huang Q., Xiao J., Chen L. (2023). Enhancement of bioavailability and bioactivity of diet-derived flavonoids by application of nanotechnology: a review. Crit Rev Food Sci Nutr.

[16] Hua S., Wu S.Y. (2013). The use of lipid-based nanocarriers for targeted pain therapies. Front Pharmacol.

[17] Bozzuto G., Molinari A. (2015). Liposomes as nanomedical devices. Int J Nanomedicine.

[18] Sipos E., Szabó Z.I., Rédai E., Szabó P., Sebe I., Zelkó R. (2016). Preparation and characterization of nanofibrous sheets for enhanced oral dissolution of nebivolol hydrochloride. J Pharm Biomed Anal.

[19] Fazil M., Md S., Haque S. (2012). Development and evaluation of rivastigmine loaded chitosan nanoparticles for brain targeting. European Journal of Pharmaceutical Sciences.

[20] Abdel-Moneim A., El-Shahawy A., Yousef A.I., Abd El-Twab S.M., Elden Z.E., Taha M. (2020). Novel polydatin-loaded chitosan nanoparticles for safe and efficient type 2 diabetes therapy: In silico, in vitro and in vivo approaches. Int J Biol Macromol.

[21] Atoufi Z., Kamrava S.K., Davachi S.M. (2019). Injectable PNIPAM/Hyaluronic acid hydrogels containing multipurpose modified particles for cartilage tissue engineering: Synthesis, characterization, drug release and cell culture study. Int J Biol Macromol.

[22] Kuo Y.-C., Chung J.-F. (2011). Physicochemical properties of nevirapine-loaded solid lipid nanoparticles and nanostructured lipid carriers. Colloids Surf B Biointerfaces.

[23] Emam H.E., El-Shahat M., Allayeh A.K., Ahmed H.B. (2023). Functionalized starch for formulation of graphitic carbon nanodots as viricidal/anticancer laborers. Biocatal Agric Biotechnol.

[24] Ahmed H.B., El-Shahat M., Allayeh A.K., Emam H.E. (2023). Maillard reaction for nucleation of polymer quantum dots from chitosan-glucose conjugate: Antagonistic for cancer and viral diseases. Int J Biol Macromol.

[25] Gupta A, Shrman K, Kushwaha G, Goyal G, Singh G, Mansoori MS. Hepatoprotective activity of silymarin against paracetamol induced liver toxicity in albino rats 2023.

[26] Bo L., Liu Y., Jia S. (2018). Metabonomics analysis of quercetin against the nephrotoxicity of acrylamide in rats. Food Funct.

[27] Siddique W., Rashid N., Asim S., Firdous A., Hanif A. (2021). Comparison of Histoprotective effect of Silymarin and Zinc Sulfate against Hepatotoxicity induced by Isoniazid and Rifampicin combination in animal model. Pakistan Journal of Medical and Health Sciences.

[28] Grancieri M, Costa NMB, Tostes M das GV, de Oliveira DS, de Carvalho Nunes L, de Nadai Marcon L, et al. Yacon flour (Smallanthus sonchifolius) attenuates intestinal morbidity in rats with colon cancer. J Funct Foods 2017;37:666–75.

[29] Schmittgen TD. Real-time quantitative PCR 2001. 10.1006/meth.2001.1260 11846607

[30] Drury R.A.B. (1983). Theory and practice of histological techniques. J Clin Pathol.

[31] Fisher K., Vuppalanchi R., Saxena R. (2015). Drug-induced liver injury. Arch Pathol Lab Med.

[32] Nolfi-Donegan D., Braganza A., Shiva S. (2020). Mitochondrial electron transport chain: Oxidative phosphorylation, oxidant production, and methods of measurement. Redox Biol.

[33] Grünig D., Duthaler U., Krähenbühl S. (2018). Effect of toxicants on fatty acid metabolism in HepG2 cells. Front Pharmacol.

[34] Farrell G.C., Larter C.Z. (2006). Nonalcoholic fatty liver disease: from steatosis to cirrhosis. Hepatology.

[35] Mostafa E.M.A., Tawfik A.M., Abd-Elrahman K.M. (2022). Egyptian perspectives on potential risk of paracetamol/acetaminophen-induced toxicities: Lessons learnt during COVID-19 pandemic. Toxicol Rep.

[36] Offor S.J., Amadi C.N., Chijioke-Nwauche I., Manautou J.E., Orisakwe O.E. (2022). Potential deleterious effects of paracetamol dose regime used in Nigeria versus that of the United States of America. Toxicol Rep.

[37] Aboraya D.M., El Baz A., Risha E.F., Abdelhamid F.M. (2022). Hesperidin ameliorates cisplatin induced hepatotoxicity and attenuates oxidative damage, cell apoptosis, and inflammation in rats. Saudi J Biol Sci.

[38] Ding K., Jia H., Jiang W., Qin Y., Wang Y., Lei M. (2023). A double-edged sword: focusing on potential drug-to-drug interactions of quercetin. Revista Brasileira De Farmacognosia.

[39] Yang D., Wang T., Long M., Li P. (2020). Quercetin: its main pharmacological activity and potential application in clinical medicine. Oxid Med Cell Longev.

[40] Janeczko M., Gmur D., Kochanowicz E., Górka K., Skrzypek T. (2022). Inhibitory effect of a combination of baicalein and quercetin flavonoids against Candida albicans strains isolated from the female reproductive system. Fungal Biol.

[41] Ferreira-Silva M., Faria-Silva C., Carvalheiro M.C. (2022). Quercetin liposomal nanoformulation for ischemia and reperfusion injury treatment. Pharmaceutics.

[42] Rayaprolu B.M., Strawser J.J., Anyarambhatla G. (2018). Excipients in parenteral formulations: selection considerations and effective utilization with small molecules and biologics. Drug Dev Ind Pharm.

[43] Vargason A.M., Anselmo A.C., Mitragotri S. (2021). The evolution of commercial drug delivery technologies. Nat Biomed Eng.

[44] Liu P., Chen G., Zhang J. (2022). A review of liposomes as a drug delivery system: current status of approved products, regulatory environments, and future perspectives. Molecules.

[45] Park S.N., Lee M.H., Kim S.J., Yu E.R. (2013). Preparation of quercetin and rutin-loaded ceramide liposomes and drug-releasing effect in liposome-in-hydrogel complex system. Biochem Biophys Res Commun.

[46] Li J., Li Z., Gao Y. (2021). Effect of a drug delivery system made of quercetin formulated into PEGylation liposomes on cervical carcinoma in vitro and in vivo. J Nanomater.

[47] Munot N., Kandekar U., Giram P.S., Khot K., Patil A., Cavalu S. (2022). A comparative study of quercetin-loaded nanocochleates and liposomes: formulation, characterization, assessment of degradation and in vitro anticancer potential. Pharmaceutics.

[48] Arzani G., Haeri A., Daeihamed M., Bakhtiari-Kaboutaraki H., Dadashzadeh S. (2015). Niosomal carriers enhance oral bioavailability of carvedilol: effects of bile salt-enriched vesicles and carrier surface charge. Int J Nanomedicine.

[49] Danaei M., Dehghankhold M., Ataei S. (2018). Impact of particle size and polydispersity index on the clinical applications of lipidic nanocarrier systems. Pharmaceutics.

[50] Song Z., Yin J., Xiao P. (2021). Improving breviscapine oral bioavailability by preparing nanosuspensions, liposomes and phospholipid complexes. Pharmaceutics.

[51] Hu R., Zheng H., Cao J., Davoudi Z., Wang Q. (2017). Synthesis and in vitro characterization of carboxymethyl chitosan-CBA-doxorubicin conjugate nanoparticles as pH-sensitive drug delivery systems. J Biomed Nanotechnol.

[52] El-Shahawy A.A.G., Abdel-Moneim A., Ebeid A.S.M., Eldin Z.E., Zanaty M.I. (2021). A novel layered double hydroxide-hesperidin nanoparticles exert antidiabetic, antioxidant and anti-inflammatory effects in rats with diabetes. Mol Biol Rep.

[53] Santiago B. (2022). Comparison of gold nanoparticle size using different measurement techniques and protocols. Characterization and Application of Nanomaterials.

[54] Benoudjit F., Maameri L., Ouared K., History A. (2020). Evaluation of the quality and composition of lemon (Citrus limon) peel essential oil from an Algerian fruit juice industry. Algerian Journal of Environmental Science and Technology December Edition.

[55] Khafagy E.-S., Almutairy B.K., Abu Lila A.S. (2023). Tailoring of novel bile salt stabilized vesicles for enhanced transdermal delivery of simvastatin: a new therapeutic approach against inflammation. Polymers (basel).

[56] Mazyed E.A., Helal D.A., Elkhoudary M.M., Abd Elhameed A.G., Yasser M. (2021). Formulation and optimization of nanospanlastics for improving the bioavailability of green tea epigallocatechin gallate. Pharmaceuticals.

[57] Ali SK, Al-Akkam EJ. Bilosomes as Soft Nanovesicular Carriers for Ropinirole Hydrochloride: Preparation and In-vitro Characterization. Iraqi Journal of Pharmaceutical Sciences (P-ISSN 1683-3597 E-ISSN 2521-3512) 2023;32:177–87.

[58] He Y., Luo L., Liang S., Long M., Xu H. (2019). Influence of probe-sonication process on drug entrapment efficiency of liposomes loaded with a hydrophobic drug. International Journal of Polymeric Materials and Polymeric Biomaterials.

[59] Mohammadi M., Ghanbarzadeh B., Hamishehkar H. (2014). Formulation of nanoliposomal vitamin D3 for potential application in beverage fortification. Adv Pharm Bull.

[60] Ong S.G.M., Ming L.C., Lee K.S., Yuen K.H. (2016). Influence of the encapsulation efficiency and size of liposome on the oral bioavailability of griseofulvin-loaded liposomes. Pharmaceutics.

[61] Ramli NA, NORA’AINI ALI, Hamzah S. Physicochemical characterization of quercetin-loaded liposomes prepared by sonication for functional food application. J Sustain Sci Manag 2020;15:15–27.

[62] Davoodi P., Lee L.Y., Xu Q. (2018). Drug delivery systems for programmed and on-demand release. Adv Drug Deliv Rev.

[63] Wei X., Yang D., Xing Z. (2021). Quercetin loaded liposomes modified with galactosylated chitosan prevent LPS/D-GalN induced acute liver injury. Materials Science and Engineering: C.

[64] Trucillo P. (2021). Drug carriers: Classification, administration, release profiles, and industrial approach. Processes.

[65] Saraswat A.L., Maher T.J. (2020). Development and optimization of stealth liposomal system for enhanced in vitro cytotoxic effect of quercetin. J Drug Deliv Sci Technol.

[66] Abdel-Azeem AS, Hegazy AM, Ibrahim KS, Farrag A-RH, El-Sayed EM. Hepatoprotective, antioxidant, and ameliorative effects of ginger (Zingiber officinale Roscoe) and vitamin E in acetaminophen treated rats. J Diet Suppl 2013;10:195–209. 10.3109/19390211.2013.822450 23927622

[67] Ramachandran A, Jaeschke H. Acetaminophen. Liver Pathophysiology, Elsevier; 2017, p. 101–12.

[68] Ozer J., Ratner M., Shaw M., Bailey W., Schomaker S. (2008). The current state of serum biomarkers of hepatotoxicity. Toxicology.

[69] El Faras A.A., Elsawaf A.L. (2017). Hepatoprotective activity of quercetin against paracetamol-induced liver toxicity in rats. Tanta Medical Journal.

[70] Thapa B.R., Walia A. (2007). Liver function tests and their interpretation. The Indian Journal of Pediatrics.

[71] Jaeschke H., Knight T.R., Bajt M.L. (2003). The role of oxidant stress and reactive nitrogen species in acetaminophen hepatotoxicity. Toxicol Lett.

[72] Kanchana N., Sadiq A.M. (2011). Hepatoprotective effect of Plumbago zeylanica on paracetamol induced liver toxicity in rats. Int J Pharm Pharm Sci.

[73] Islam M.T., Quispe C., Islam M.A. (2021). Effects of nerol on paracetamol-induced liver damage in Wistar albino rats. Biomedicine & Pharmacotherapy.

[74] Pawlikowska-Pawlęga B., Gruszecki W.I., Misiak L. (2007). Modification of membranes by quercetin, a naturally occurring flavonoid, via its incorporation in the polar head group. Biochimica et Biophysica Acta (BBA)-Biomembranes.

[75] Ahmed O.M., Elkomy M.H., Fahim H.I. (2022; 2022.). Rutin and quercetin counter doxorubicin-induced liver toxicity in Wistar rats via their modulatory effects on inflammation, oxidative stress, apoptosis, and Nrf2. Oxid Med Cell Longev.

[76] Massart J., Begriche K., Fromenty B. (2021). Cytochrome P450 2E1 should not be neglected for acetaminophen-induced liver injury in metabolic diseases with altered insulin levels or glucose homeostasis. Clin Res Hepatol Gastroenterol.

[77] MacFie C., Wall E., Ash S. (2009). Paracetamol overdose presenting with hyperglycaemia, acidosis and ketonuria in a non-diabetic patient. Acute Med.

[78] Yue S., Zhou H.M., Zhu J.J. (2015). Hyperglycemia and liver ischemia reperfusion injury: a role for the advanced glycation endproduct and its receptor pathway. American Journal of Transplantation.

[79] M Eid H, S Haddad P. The antidiabetic potential of quercetin: underlying mechanisms. Curr Med Chem 2017;24:355–64. 10.2174/0929867323666160909153707 27633685

[80] Long X., Song J., Zhao X. (2020). Silkworm pupa oil attenuates acetaminophen‐induced acute liver injury by inhibiting oxidative stress‐mediated NF‐κB signaling. Food Sci Nutr.

[81] Jiang W.-P., Deng J.-S., Huang S.-S. (2021). Sanghuangporus sanghuang mycelium prevents paracetamol-induced hepatotoxicity through regulating the MAPK/NF-κB, Keap1/Nrf2/HO-1, TLR4/PI3K/Akt, and CaMKKβ/LKB1/AMPK pathways and suppressing oxidative stress and inflammation. Antioxidants.

[82] El Fadil H.A., Edress N., Khorshid N., Amin N. (2019). Protective Impact of Curcumin against Paracetamol-Induced Hepatotoxicity in Rats. International Journal of Pharmaceutical Research & Allied. Sciences.

[83] Berktas O.A., Peker E.G.G. (2022). Protective effects of Prunus laurocerasus extracts against paracetamol-induced hepatotoxicity. Nutrition and Food Processing.

[84] Surapaneni K.M., Jainu M. (2014). Comparative effect of pioglitazone, quercetin and hydroxy citric acid on the status of lipid peroxidation and antioxidants in experimental non-alcoholic steatohepatitis. J Physiol Pharmacol.

[85] Hosseini A., Razavi B.M., Banach M., Hosseinzadeh H. (2021). Quercetin and metabolic syndrome: a review. Phytotherapy Research.

[86] Shen X.-L., Guo Y.-N., Lu M.-H. (2023). Acetaminophen-induced hepatotoxicity predominantly via inhibiting Nrf2 antioxidative pathway and activating TLR4-NF-κB-MAPK inflammatory response in mice. Ecotoxicol Environ Saf.

[87] Luo G., Huang L., Zhang Z. (2023). The molecular mechanisms of acetaminophen-induced hepatotoxicity and its potential therapeutic targets. Exp Biol Med.

[88] McGill M.R., Hinson J.A. (2020). The development and hepatotoxicity of acetaminophen: reviewing over a century of progress. Drug Metab Rev.

[89] Kitteringham N.R., Powell H., Clement Y.N. (2000). Hepatocellular response to chemical stress in CD-1 mice: induction of early genes and γ-glutamylcysteine synthetase. Hepatology.

[90] Laddha A.P., Wu H., Manautou J.E. (2024). Deciphering Acetaminophen-Induced Hepatotoxicity: the crucial Role of Transcription Factors like Nuclear factor Erythroid 2–Related factor 2 as Genetic Determinants of Susceptibility to Drug-Induced Liver Injury. Drug Metabolism and Disposition.

[91] Tebay L.E., Robertson H., Durant S.T. (2015). Mechanisms of activation of the transcription factor Nrf2 by redox stressors, nutrient cues, and energy status and the pathways through which it attenuates degenerative disease. Free Radic Biol Med.

[92] Zhang D.D., Chapman E. (2020). The role of natural products in revealing NRF2 function. Nat Prod Rep.

[93] Chou A.-H., Lee H.-C., Liao C.-C., Yu H.-P., Liu F.-C. (2023). ERK/NF-kB/COX-2 signaling pathway plays a key role in curcumin protection against acetaminophen-induced liver injury. Life.

[94] Múnera-Rodríguez A.M., Leiva-Castro C., Sobrino F., López-Enríquez S., Palomares F. (2024). Sulforaphane-mediated immune regulation through inhibition of NF-kB and MAPK signaling pathways in human dendritic cells. Biomedicine & Pharmacotherapy.

[95] Ren S., Leng J., Xu X.-Y. (2019). Ginsenoside Rb1, a major saponin from Panax ginseng, exerts protective effects against acetaminophen-induced hepatotoxicity in mice. Am J Chin Med (gard City N y).

[96] Bourdi M., Korrapati M.C., Chakraborty M., Yee S.B., Pohl L.R. (2008). Protective role of c-Jun N-terminal kinase 2 in acetaminophen-induced liver injury. Biochem Biophys Res Commun.

[97] Yi R.-K., Song J.-L., Lim Y.-I., Kim Y.-K., Park K.-Y. (2015). Preventive effect of the Korean traditional health drink (Taemyeongcheong) on acetaminophen-induced hepatic damage in ICR mice. Prev Nutr Food Sci.

